# Clinical Characteristics and Rehabilitation Strategies for the Stomatognathic System Disturbances in Patients with Stroke: A Systematic Review

**DOI:** 10.3390/ijerph20010657

**Published:** 2022-12-30

**Authors:** Mónica Zapata-Soria, Irene Cabrera-Martos, Laura López-López, Araceli Ortiz-Rubio, María Granados-Santiago, Izarbe Ríos-Asín, Marie Carmen Valenza

**Affiliations:** 1 Department of Physiotherapy, University of Granada, 18016 Granada, Spain; 2 Department of Nursing, University of Granada, 52071 Granada, Spain

**Keywords:** systematic review, stomatognathic system, assessment, rehabilitation

## Abstract

**Background:** Understanding the stomatognathic system disturbances is key to diagnosing them early and implementing rehabilitation approaches to promote functional recovery. The objective of this study was to systematically review all published data that examined the assessment and rehabilitation strategies for the stomatognathic system disturbances in patients with stroke. **Methods:** Five databases (i.e., PubMed/MEDLINE, Scopus, Science Direct, Web of Science, and PEDro), were screened for manuscripts that included the assessment and rehabilitation strategies for stomatognathic system disturbances. The methodological quality was evaluated using the Mixed Methods Appraisal Tool. **Results:** Sixteen articles were included in this systematic review. The most frequently reported symptoms in patients with stroke included stiffness and thickness of the masseter muscle on the affected side and suprahyoid muscles; facial muscles’ asymmetry and weakness; temporomandibular disorders; and a reduced maximum lip force, tongue pressure, and saliva flow rate. The rehabilitation strategies more frequently reported included exercises directed to the jaw, temporomandibular joint, tongue, and neck. The mean score for methodological quality was 85%. **Conclusion:** The stomatognathic system disturbances are frequently reported among patients with stroke, leading to dysfunction in masticatory performance or swallowing. More studies on interventions for stomatognathic system disturbances are required before conclusions may be drawn. **Key Practitioner Message:** This systematic review has clinical implications for rehabilitation practices, given that the results may help to develop early assessment and rehabilitation strategies for stomatognathic disturbances in patients with stroke.

## 1. Introduction

Stroke is one of the leading causes of acquired disability worldwide [1], causing frequent medical complications and long-term sequelae [2]. Depending on the size of the lesions and the affected cerebral structures (i.e., cortical areas, central nervous system pathways, or motor-neuron pools of the cranial nerves in the brain stem), post-stroke sensorimotor deficits related to the stomatognathic system may be present [3,4]. The stomatognathic system is a functional complex formed by structures located within the oral and craniofacial cavities [5], including skeletal components, head and neck muscles, ligaments, soft tissues, the temporomandibular joint, dental arcs, salivary glands, and masticatory muscles [6,7].

Previous studies have reported changes in the stomatognathic functions in patients after a stroke including decreased bite force and quality of mastication, reduced lip force, and asynchronous movements of the tongue [2,4]. These deficits may have a negative impact on functions such as chewing efficiency, swallowing, facial expressivity, and phonation [8,9,10].

Understanding the stomatognathic system disturbances is key to diagnosing them early and implementing rehabilitation approaches to promote functional recovery [11]. Previous studies have shown different interventions involving the stomatognathic system’s structures and functions [12,13]. In addition, Shimmel et al. [14] reported that orofacial symptoms seem not to improve without a specific rehabilitation approach. However, no previous review has addressed the clinical characteristics and rehabilitation strategies for stomatognathic system disturbances after a stroke. Thus, this systematic review aims to evaluate the disturbances of the stomatognathic system, and the rehabilitation strategies developed in patients with stroke.

## 2. Methods

### 2.1. Registration and Protocol

This systematic review was reported following the Preferred Reporting Items for Systematic Reviews and Meta-Analyses (PRISMA-P) guidelines [15]. It was previously registered in the International Prospective Register of Systematic Reviews (PROSPERO), registry number CRD42020221806.

The following databases were searched from database inception until March 2022: PubMed/MEDLINE, Scopus, Science Direct, Web of Science, and PEDro. The search strategy used was as follows: ((Stomatognathic System OR stomatognathic OR temporomandibular joint OR cheek OR facial muscles OR jaw OR masticatory muscles OR mouth OR pharynx) AND (assessment OR outcome OR evaluation OR stomatognathic system abnormalities OR temporomandibular joint disorder OR temporomandibular joint dysfunction OR malocclusion OR mastication OR jaw abnormalities OR mouth abnormalities OR facial paralysis OR tooth abnormalities) AND (stroke or cerebrovascular accident or cerebrovascular disease OR brain vascular accidents OR cerebral strokes OR brain ischemia OR cerebral ischemia OR hemiplegia OR hemiparesis OR hemorrhagic stroke OR intracerebral hematoma OR intracerebral hemorrhage OR lacunar stroke)).

### 2.2. Search Strategy

The studies were included according to the following eligibility criteria. The inclusion criteria were people with stroke over 18 years of age, studies reporting outcomes for stomatognathic system assessment, and/or rehabilitation strategies for stomatognathic system disturbances. The structures of the stomatognathic system include temporomandibular joints, jaw and mandible, muscle tissues and tendons, dental arches, salivary glands, as well as the hyoid bone and the muscles that connect the latter to the scapula and the sternum and the muscles of the neck [16].

The exclusion criteria were participants with any neurological disease other than stroke; articles not published in English, French, or Spanish; and no full-text access. Editorials, discussion papers, conference abstracts, reviews, and abstracts were also excluded.

### 2.3. Selection Strategy

All the citations were imported into Mendeley, and duplicate records were removed before the screening. Two independent reviewers assessed all results after the removal of duplicates, using the information provided in the title and abstract. Then, full texts were reviewed, and the data extraction was completed by two authors. A third author was available to resolve any discrepancies.

### 2.4. Study Quality Assessment

The methodological quality was evaluated by the Mixed Methods Appraisal Tool (MMAT) version 2018: a critical appraisal tool designed for the appraisal stage of systematic mixed studies reviews [17]. The MMAT assesses the quality of different study designs, i.e., qualitative, quantitative, and mixed methods studies. 

This tool includes criteria for appraising the methodological quality of five categories of studies: (a) qualitative studies, (b) randomized controlled trials, (c) non-randomized studies, (d) quantitative descriptive studies, and (e) mixed methods studies. For each study category, the tool includes two screening questions and five questions targeted to evaluate the specific characteristics of each category.

Each criterion is rated on a categorical scale: yes, no, and cannot tell. A quantitative score was calculated using the following formula [17]: [(number of “yes” responses scored as 1 divided by the number of criteria) × 100]. The methodological quality assessment was carried out by two reviewers and any discrepancies were resolved by contacting a third author.

Additionally, the risk of bias for the randomized controlled trials included was also assessed with version 2 of the Cochrane risk-of-bias tool (RoB-2) [18]: This tool consists of five domains that focus on the randomization process, deviations from the intended interventions, missing outcome data, measurement of the outcome, and the selection of the reported result. The studies were interpreted as having a high, low, or unclear risk of bias.

## 3. Results

**Figure 1:**
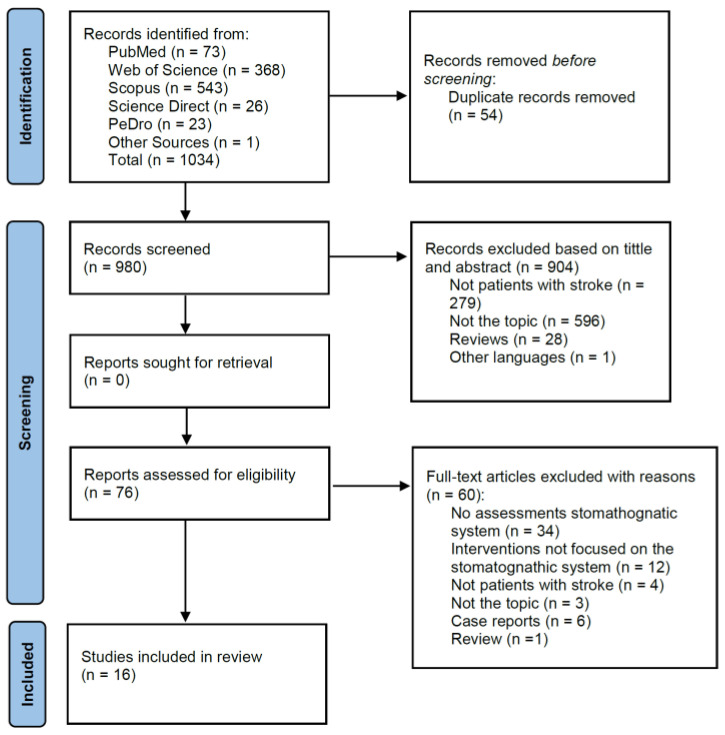
Flow chart.

A total of 1034 studies were identified in the search strategy, of which 16 were included in the systematic review [3,4,7,12,13,19,20,21,22,23,24,25,26,27,28,29] (Figure 1).

The first author, inclusion and exclusion criteria, total sample, mean age, gender, time since stroke, and setting are included in Table 1.

Table 2 summarizes the design, objective, the structures of the stomatognathic system assessed, outcomes related to the stomatognathic system, characteristics of the rehabilitation strategies and frequency and intervention duration, and the main results regarding the stomatognathic system disturbances and rehabilitation strategies obtained in the included studies.

### 3.1. Structural Changes in the Stomatognathic System in Patients with Stroke

The stomatognathic system structures assessed included the tongue, orbicularis muscles, jaw, masticatory muscles, lips, cheeks, teeth, suprahyoid muscles, hyoid bone, temporomandibular joint, and muscles of the neck [3,4,7,12,13,19,20,21,22,23,24,25,26,27,28,29].

Several studies demonstrated post-stroke dysfunction in facial muscles [7,12,23,29]. Schimmel et al. found higher facial asymmetry in the lower face in stroke patients with reduced muscle tonus and weakness in orbicularis, zygomaticus major, and risorius muscles [23]. Umay et al. observed significantly lower muscle action potentials in amplitudes of orbicularis and masseter muscles [12]. Song et al. showed that the masseter muscle hardness on the unaffected side was significantly greater than on the affected side [29]. The masseter thickness was evaluated by other authors with similar results showing that the masseter muscle on the affected side was thinner than on the non-affected side [3,24]. In addition, a lower pressure pain threshold in the masseter muscle was evidenced by Altvater Ramos et al. [27] and Dursun et al. [7], who observed a sensorimotor loss in facial muscles in patients with stroke. In addition, mylohyoid and digastric muscles were evaluated by Choi et al. [28], who observed a decrease in the thickness of these muscles. 

The temporomandibular disorders (TMD) were frequently reported among post-stroke patients in the studies included. They reported that patients with stroke had more of a tendency to develop temporomandibular joint disorders [7,13,27]. The studies conducted by Altvater Ramos, Dursun, and Oh et al. showed limitations in the range of motion of the temporomandibular joint, reduced disk displacement, and a decreased craniomandibular index [7,13,27]. Specifically, the study conducted by Dursun et al. showed that stroke patients presented higher scores on the Fonseca Questionnaire [7]. 

Dysfunctions in the temporomandibular joint have shown to be frequently related to limitations on cervical mobility and postural abnormality [22,27]. Two studies in this review evaluated cervical mobility, showing that patients with stroke have poor posture with head and neck alignment dysfunction [22,27]. Moreover, Altvater Ramos et al. assessed the neck range of motion using a fleximeter evaluation and showed that all the patients had a decreased amplitude of the cervical spine movements [27].

### 3.2. Stomatognathic System Dysfunctions in Patients with Stroke

The most reported affected functions in patients with stroke in the studies included in this systematic review were masticatory performance [19,24,26,29], and swallowing [12,13,25,26,28].

Four studies demonstrated a decrease in masticatory performance [19,24,26,29]. Song et al. [29] found that the masticatory performance of the masseter muscle was significantly greater on the unaffected side than on the affected side. Furthermore, a negative correlation was found between masseter muscle stiffness and masticatory performance [29]. Schimmel et al. [26] showed lower chewing efficiency in stroke patients and exhibited a possible relationship between oral sensitivity and masticatory performance. In addition, two studies showed that masticatory efficiency was severely affected after a stroke [19,24]. 

Three of the studies included examined the bite function in patients with stroke [21,22,24]. One study reported that the maximum bite force between both sides was not significantly different in both groups [19]. However, Kawasaka et al. showed lower bite force between the hemi-plegic side in stroke patients and the mean of bilateral sides in the control group [21]. In addition, Schimmel et al. [24] observed that the maximum bite force was not significantly different between both sides and between the experimental and control groups. 

The swallowing function is characterized by a complex and coordinated activation of many of the stomatognathic system structures. The swallowing capacity, ability, and bolus transition were evaluated in different studies [13,20,25]. Umay et al. reported longer swallowing intervals in the patients with stroke compared to the healthy control group [12]. Furthermore, salivary secretion was assessed in two studies showing a lower flow rate in patients with stroke [19,21].

### 3.3. Rehabilitation Strategies for Stomatognathic System Disturbances

Five of the selected studies in this review reported an intervention focused on stomatognathic system disturbances [4,12,13,25,28]. Most of the studies included exercises (e.g., mobility, resistance training, breathing, and postural exercises) including the jaw, temporomandibular joint, tongue, and neck [4,13,25,28]. One study used a non-invasive electrical stimulation to the masseter muscle added to standard rehabilitation [12]. The protocol used, training duration, frequency of training, and study period were heterogeneous.

Two of the studies [4,13] included measures about temporomandibular joint mobility and the craniomandibular index. Groups that underwent mobility exercises in combination with breathing and posture or stomatognathic alignment exercises showed a significant improvement after the intervention. The study of Oh et al. also improved swallowing function [13].

Choi et al. [28] compared two interventions added to the traditional dysphagia treatment, one focused on jaw opening exercises and the other on head lift exercises. Both interventions exhibited similar results, with a statistically significant increase in the thickness of the digastric and mylohyoid muscles, and anterior and superior movement of the hyoid bone [28]. The study of Steele et al. focused on tongue training comparing two training protocols, tongue-pressure strength and accuracy training, and tongue-pressure profile training including real swallows rather than pressure generation tasks in isolation. The study suggested that there was a significant treatment effect on tongue strength and swallowing with similar effects in both groups [25]. 

Only the study of Umay et al. [12] used intermittent galvanic stimulation to the masseter muscle added to standard dysphagia and swallowing rehabilitation, obtaining a significant improvement in muscle dynamic activity and significant recovery in swallowing.

### 3.4. Methodological Quality

The methodological quality is included in Table 3. The 16 included studies were categorized as quantitative research, 4 (25%) were randomized controlled trials, 11 (68, 75) were non-randomized studies, and 1 (6, 25%) was descriptive research. Overall, the methodological quality of the 16 studies was 85%, and varied from 40% (*n* = 1) to 100% (*n* = 9).

**Figure 2:**
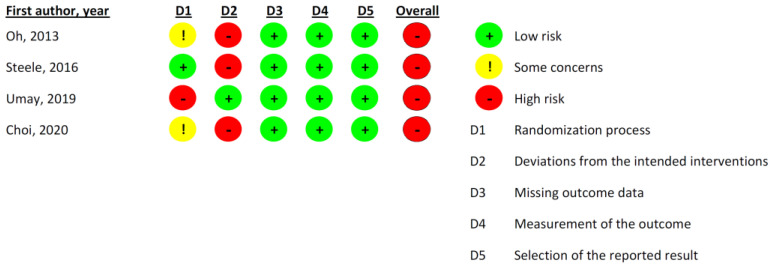
Risk of bias [12,13,25,28].

The risk of bias in the randomized controlled trials included was assessed with the Cochrane RoB-2 tool and is included in Figure 2. It showed a high risk for all the studies included. The most frequent domain showing a high risk of bias was the deviations from the intended interventions. All the studies presented a low risk of bias in the missing outcome data, measurement of the outcome, and selection of the reported result domains.

## 4. Discussions

This systematic review aimed to provide detailed information on the clinical characteristics and rehabilitation strategies for stomatognathic system disturbances in patients with stroke. The results show that patients with stroke frequently show increased stiffness and thickness of the masseter muscle on the affected side and suprahyoid muscles. Facial muscles also show increased asymmetry, especially lower facial muscles, where weakness may be increased, and muscle action potential amplitudes reduced. Other changes such as sensitivity to pressure pain, tactile detection, or two-point discrimination may be reduced. In addition, a high number of patients with stroke have TMD and a reduced maximum lip force, tongue pressure, and saliva flow rate. These structural changes are linked to reduced masticatory performance, chewing efficiency, and an increased duration of the oral phase of swallowing. Regarding the rehabilitation strategies for stomatognathic disturbances, there is limited evidence. Most of the studies incorporated exercises (e.g., mobility, resistance training, breathing, and postural exercises) including the jaw, temporomandibular joint, tongue, and neck.

In this review, various studies showed that masticatory performance and chewing can be affected after a stroke [21,22,24,29]. Chewing dysfunction is common in most hospitalized patients with stroke [30,31]. The study of Song Yu et al. showed that masticatory efficiency can be affected one year after stroke [29]. In addition, Schimmel et al. also observed lower chewing efficiency two years after a stroke [24].

The temporomandibular joint allows chewing, swallowing, and speaking [32]. Stroke patients may have temporomandibular joint disorders with a dislocation of the temporomandibular joint because of limited jaw movement and pain [33,34]. In this review, some studies [4,7,13,26] reported that dysfunction of the temporomandibular joint is associated with mouth opening limitation and asymmetrical mandibular movements. Although the literature that explores the impact of TMD on phonation in patients with stroke is limited, it has been previously shown that these disorders are related to a reduction in mandibular opening and retrusion movements during speech [35]. Oh and Yilmaz et al. proposed a rehabilitation program focused on the temporomandibular joint including mobility exercises in combination with breathing and posture or stomatognathic alignment exercises showing significant improvement after the intervention [4,13]. According to Shaffer et al. [36], clinicians should design rehabilitation programs that address both symptom reduction and oral function considering patient-specific impairments. Durham et al. [37] indicated that persistent TMD can be associated with other chronic pain conditions including migraine or widespread pain. In this sense, early management with education and counseling is highly effective.

According to different studies [21,22,24], lower bite force can affect the eating process in patients with stroke. Kawasaka et al. demonstrated that the most affected side showed reduced values, but there were no significant differences from the mean of bilateral sides of the participants in the control group [21]. However, Schimmel et al. [22,24] observed that the maximum bite force was reduced in the group of patients with stroke without differences between both sides. In this sense, Miles and Nordstrom showed that both cortical hemispheres are involved in masticatory control and the muscles on both sides are usually used together [38]. Additionally, the weakness of facial muscles can influence masticatory performance [39,40]. Moreover, a previous study reported that a lower bite force in patients with stroke could be related to a change in diet or disuse due to muscular atrophy [41]. 

In this systematic review, eight studies showed impairments in facial muscles [3,7,12,23,24,27,28,29]. Muscles of facial expression assist in speech articulation, emotions, expressions, and bolus preparation [42]. Moreover, the facial muscles are key for social interaction, including speech and non-verbal communication [43]. Different structures of the nervous system are involved in the control of facial expressions, such as the primary motor cortex, the ventral lateral premotor cortex, and the supplementary motor for voluntary control, and the cingulate cortical areas are important for emotional expression [44]. Orbicularis, masseter, zygomaticus, risorius, and temporalis muscles were evaluated and showed lower amplitudes, reduced tonus, asymmetry, and thickness [3,7,12,23,24,27,28,29]. In this line, a previous systematic review found evidence of the loss of facial muscle mass after a stroke [45]. Limited evidence was found for rehabilitation strategies on facial muscles. A recent systematic review described that among these interventions the use of an oral screen, neuromuscular electrical stimulation, mirror therapy, or exercises are included [46]. In this review, the study conducted by Umay et al. [12] showed promising results in the use of intermittent galvanic stimulation to the masseter muscle added to standard dysphagia rehabilitation, obtaining a significant improvement in muscle dynamic activity and significant recovery in swallowing. A previous study assessed the effects of sensory-level electrical stimulation treatment combined with conventional dysphagia rehabilitation in the pediatric population with cerebral palsy, showing that sensory-level electrical stimulation might be a useful and safe therapeutic modality to improve oropharyngeal symptoms, symptom severity, and dysphagia [47]. 

Choi et al. demonstrated lower thickness in digastric and mylohyoid muscles [28], which may affect the kinematics of the hyoid bone [4]. Although there is limited research involving rehabilitation strategies, Choi et al. [28] compared two interventions: one focused on jaw opening exercises, and the other one on head lift exercises. Both achieve a significant increase in the thickness of the digastric and mylohyoid muscles and anterior and superior movement of the hyoid bone.

Cortical lesions that affect precentral gyrus may produce impairments of the motor and sensory functions of the face, lips, and tongue [14]. A reduced tongue pressure and lip force can influence the masticatory performance [48,49]. The tongue is involved in functions such as the oral preparatory stage, oral propulsive stage, and food processing [48]. Reduced tongue pressure deteriorates the ability to control swallowing [49]. Daniels et al. showed that subcortical lesions disconnect cortical regions from oral control and coordination in swallowing, thus producing lingual discoordination during swallowing [50]. In addition, the sensitivity of the tongue may be affected [26]. Steele et al. [25] compared two training protocols including a tongue-pressure exercise, resulting in an improvement in strength and swallowing. In this line, the study of Svensson et al. [51] suggested that the specific and reversible plasticity of the corticomotor excitability related to tongue muscle control can be induced when humans learn to perform a novel tongue task successfully. Namiki et al. [52] developed tongue-pressure resistance training in healthy participants achieving an improvement in tongue strength, dexterity, both anterior and superior hyoid elevation, and swallowing functions.

Lips are also involved in the oral phase, being crucial to contain liquid or saliva in the oral cavity or to prevent drooling [53]. Different studies in this review demonstrated a reduced maximum lip force and salivary flow rate after stroke. No rehabilitation strategies have been developed that focus on lip force and salivary flow.

The impairments of the facial muscles, lips, tongue, and jaw can influence the development of speech disorders among stroke patients [54]. The study conducted by De Cock et al. examined the speech characteristics, type of dysarthria, and severity in patients with acute stroke. They found that the imprecise articulation of consonants, harsh voice quality, and audible inspiration were the most frequently observed speech characteristics [55]. Their results showed that maximum phonation time, maximum loudness, and speech intelligibility were impaired in patients with acute stroke. Robertson proposed that a rehabilitation strategy including a program focused on orofacial muscle movement and articulation is effective in improving motor speech overall and in increasing intelligibility after a stroke [56]. In this line, a previous systematic review with meta-analysis concluded that an alternating and sequential motion rate and maximum phonation time significantly improve after a speech rehabilitative treatment in patients with stroke [54]. 

### Limitations

Our review included studies conducted in patients with stroke, without considering the phase. In addition, the small number of studies focused on rehabilitation strategies limits our ability to analyze the results obtained. In this sense, caution should be applied when generalizing the results, as heterogeneity is present in the population, design, and outcome measures. Most of the studies included presented a small sample size or specific inclusion criteria, which limit the generalization of the results. In addition, a meta-analysis of the results of the follow-up was not possible. Accordingly, caution is needed when interpreting the results and generalizing the outcomes in patients with stroke. Although we reviewed multiple electronic databases of published studies, we may have missed some trials. Given the limited number of studies focused on interventions, it was not possible to formulate specific questions for each outcome to develop a clinical practice recommendation.

## 5. Conclusions

Stomatognathic system disturbances are frequently reported among patients with stroke, leading to dysfunction in important functions such as masticatory performance or swallowing. The most frequently reported symptoms include stiffness and thickness of the masseter muscle on the affected side and suprahyoid muscles; facial muscles’ asymmetry and weakness; TMD and a reduced maximum lip force, tongue pressure, and saliva flow rate. The rehabilitation strategies more frequently reported include exercises directed to the jaw, temporomandibular joint, tongue, and neck. However, the evidence is limited. Thus, the design of optimal treatment strategies to adequately prevent deterioration in these symptoms is of relevance. This review updates the evidence and provides an overview for clinical characteristics and rehabilitation strategies for stomatognathic disturbances including the most recent trials. Future research is needed to identify the early detection strategy and the optimal protocol, frequency, duration, and intensity for maximizing functional improvements in this population.

## Figures and Tables

**Table 1:** Characteristics of the studies included in the systematic review.

First Author, Year, Reference	Inclusion Criteria	Exclusion Criteria	Sample (Total Sample, Number of Groups)	Mean Age (Per Group) ± SD (Total and Per Group)	Gender (% Women Per Group)	Time Since Stroke (Mean ± SD; Mean, Range)	Setting
kim, 2005 [19]	- Stroke - Dysphagia	- Cognitive impairment	Total: *n* = 20 2 groups: EG (*n* = 10) CG (*n* = 10)	64.6 y (50, 82)	EG: 50 CG: 50	9.8 w (2, 24 w)	Department of Rehabilitation Medicine, Clinical Research Institute, National University Hospital, Seoul, Korea
Kawasaka, 2010 [21]	- Ability to chew using the molar teeth	- Dental and oral diseases, such as gingivitis, aphthae, or inflammation of the salivary glands	Total: *n* = 60 2 groups: EG (*n* = 30) CG (*n* = 30)	EG = 61.4 ± 2.3 y CG = 63.4 ± 3.1 y	EG: 53.33 CG: 63.33	8.3 ± 2.1 mo	Kirishima Rehabilitation Center of Kagoshima, University Hospital, Japan
Schimmel, 2010 [3]	- Ischaemic or haemorrhagic stroke - Hemi-syndrome with facial palsy - No dysphagia - Ability to give informed consent and follow instructions	- No ability to understand information - Methicillin-resistant staphylococcus aureus infection	Total: *n* = 55 2 groups: EG (*n* = 31) CG (*n* = 24)	EG = 69.0 ± 12.7 y CG = 68.8 ± 10.8 y	EG: 41.9 CG: 45.8	42.3 ± 14.4 d (18, 85)	Division of Neuro-rehabilitation at the Department for Clinical Neurosciences of the University Hospitals of Geneva
Schimmel, 2011a [20]	- Ischaemic or haemorrhagic - Stroke - Hemi-facial and/or limb palsy - Ability to follow simple instructions and perform the tests	- No ability to understand the patient information - Methicillin-resistant staphylococcus aureus infection	Total: *n* = 55 2 groups: EG (*n* = 31) CG (*n* = 24)	EG = 69.0 ± 12.7 y CG = 68.8 ± 10.8 y	EG: 41.9 CG: 45.8	42.3 ± 14.4 d	Division of Neuro-rehabilitation at the Department for Clinical Neurosciences of the University Hospitals of Geneva
Schimmel, 2011b [22]	- Ischaemic or haemorrhagic stroke - Hemi-syndrome with facial palsy - Ability to follow instructions	- Infectious disease	Total: *n* = 55 2 groups: EG (*n* = 31) CG (*n* = 24)	EG = 69.0 ± 12.7 y CG = 68.8 ± 10.8 y	EG: 41.9 CG: 45.8	42.3 ± 14.4 d	Division of Neurorehabilitation of the Department of Clinical Neurosciences of the University Hospitals of Geneva, Switzerland
Schimmel, 2011c [23]	- Hemi-paresis on the face - Ability to understand the patient information and to perform various clinical tests - Free of infectious disease	- NR	Total: *n* = 49 2 groups: EG (*n* = 27) CG (*n* = 22)	EG = 68.7 ± 12.9 y CG = 69.0 ± 11.2 y	EG: 44.45 CG:45.45	43.8 ± 14.2 d	Division of Neurorehabilitation of the Department of Clinical Neurosciences of the University Hospitals of Geneva, Switzerland
Schimmel, 2013 [24]	- Stroke, hemi-syndrome with facial palsy - Consent to participate and capability to perform various clinical tests	- NR	Total: *n* = 20 2 groups: EG (*n* = 10) CG (*n* = 10)	EG: n = 64.1 ± 17.4 y CG: n = 64.4 ± 18.6 y	EG: 40 CG: 40	>6 mo	Division of Neurorehabilitation of the Department of Clinical Neurosciences of the University Hospitals of Geneva, Switzerland
Oh, 2013 [13]	- Stroke >6 mo - Decreased TMJ function > 0.13 points on the CMI - CMI and mouth opening <4.0 cm	- Orthopaedic or musculoskeletal conditions and cognitive impairment	Total: *n* = 14 2 groups: EG (*n* = 7) CG (*n* = 7)	EG = 53.71 ± 12.46 y CG = 56.14 ± 12.31 y	EG: 28.57 CG: 28.57	EG: 43.00 ± 27.90 mo CG: 13.57 ± 16.53 mo	Wonkwang University Hospital, Republic of Korea
Steele, 2016 [25]	- Recent stroke with swallowing difficulties - Tongue-palate pressure measure <40 kPa - Stage transition duration of 350 ms on liquid barium swallow during the intake	- Severe dysphagia with no functional opening of the upper esophageal sphincter - Pre-existing dysphagia or head and neck cancer	Total: *n* = 14 2 groups: TPPT (*n* = 7) TPSAT (*n* = 7)	TPPT: 74.85 TPSAT: 67.14	Male: 64.3 Female: 35.7	70 d (range 18–150)	Three stroke rehabilitation centers in Ontario, Canada
Schimmel, 2017 [26]	- Stroke patients who were able to undergo psychophysical testing - House–Brackmann ≥ 2	- Acute pain in the oro-facial sphere or an additional neuro-muscular disease - Patients with tube feed or acute risk of aspiration because of dysphagia	Total: *n* = 54 2 groups: EG (*n* = 27 CG (*n* = 27)	EG = 64.3 ± 14.1 y CG = 60.8 ± 14.3 y	EG: 70.37 CG: 62.96	EG: 31.00 ± 54.00 d	Division of Neurorehabilitation of the Department of Clinical Neurosciences of the University Hospitals of Geneva, Switzerland
Dursun, 2018 [7]	- Subacute and chronic stroke	- Systemic or congenital disease - Cooperation problems - Jaw fracture, postfacial paralysis, and orthognathic surgery	Total: *n* = 100 2 groups: EG (*n* = 50) CG (*n* = 50)	EG = 62.16 ± 11.41 y CG = 59.7 ± 9.62 y	NR	NR	Bolu Izzet Baysal Physical Therapy and Rehabilitation Education and Research Hospital. Bolu and Düzce cities.
Altvater Ramos, 2019 [27]	- MMSE > 19 - More than 3 mo since stroke	- Refusal to participate in the study, muscle hypotonia, and edentulous patients without the use of dental prosthesis	*n* = 11 1 group	55–70 y	NR	>3 mo	Physical therapy and rehabilitation center from Universidade Estadual do Norte do Paraná (UENP), Jacarezinho, Paraná
Umay, 2019 [12]	- Ischemic stroke within 20–60 d and swallowing disorders	- Malignancy, head and neck surgery, previous stroke, and respiratory distress, smoking and alcoholism, and hemorrhagic and/or bilateral stroke - Contraindication for electrical stimulation	Total: *n* = 102 2 groups: EG (*n* = 51) CG (*n* = 51)	EG = 63.68 ± 9.13 y CG = 65.41 ± 8.47 y	EG: 41.2 CG: 35.3	20–60 d	Physical Medicine and Rehabilitation Clinic, Ankara Diskapi Yildirim Beyazit Education and Research Hospital, Ankara Turkey
Yilzman, 2020 [4]	- Age 60–75, ischemic or hemorrhagic stroke and TMJD	- Patients over 75 y - Progressive neurological disease - Orofacial congenital malformation - Previous neck and head surgery	*n* = 30 1 group	68.73 ± 4.79 y	26.7	8.00 ± 2.22 mo	Physical Medicine and Rehabilitation Clinic and Rehabilitation Center Hospital, Kastamonu, Turkey
Choi, 2020 [28]	- Dysphagia - Ability to follow instructions - Ability to swallow - Liquid aspiration or penetration - Nasogastric tube - Ability to use arm - MMSE > 22	- Secondary stroke, brainstem stroke, and other neurologic diseases - Pain in the disc, cervical spine and jaw, limitations in jaw opening, cervical spine orthosis, cervical spine surgery - Myelopathy, gastrostomy tube, and problems with esophageal phase in dysphagia	Total: *n* = 21 2 groups: JOE group (*n* = 11) HLE group (*n* = 10)	JOE group: n = 63.47 ± 7.65 y HLE group: n = 61.24 ± 9.73 y	57.14	1 to 5 m	Two hospitals in South Korea
Song, 2021 [29]	- Ischemic stroke > 12 mo - Activities of daily living score (ADL) > 60 and limb function on affected side (>Brunnstrom IV)	- Age <18 or >60 y - Systemic disease or cognitive disorder - Periodontal treatment, absence of teeth, use of dentures or prosthesis - Acute oral infections - Pregnancy or breastfeeding - Smoking and alcohol or drugs affecting muscle tone consumption	*n* = 20 1 group	47.65 ± 9.16 y	50	<1 y	Luoyang Orthopaedic Hospital of Henan Province, China

d: days; CG: control group; CMI: craniomandibular index; EG: experimental group; HLE: head lift exercise; JOE: jaw opening exercise; MMSE: mini-mental state examination; mo: months; TMJD: temporomandibular joint dysfunction; TPPT: tongue-pressure profile training; TPSAT: tongue-pressure strength and accuracy training.

**Table 2:** Study details on assessment and rehabilitation interventions for stomatognathic system disturbances in patients with stroke.

First Author, Year, Reference	Design	Intervention/Control	Frequency (Min Per Sesion/Sessions Per w)/Intervention Duration (w)	Structures of the Stomatognathic System Assessed	Outcome Measures (Device, Tool)	Main Results
kim, 2005 [19]	Non-randomized study			Saliva, masticatory performance	Spitting and weight measure of salivary flow for 30 min. Total chew duration oral phase from the time of the test food.	Saliva flow rate was significantly lower in stroke patients. Stroke patients chewed for a longer time.
Kawasaka, 2010 [21]	Non-randomized study			Teeth	Modified cotton swab method for salivary secretion. Oclusal force.	Salivary secretion was reduced in cerebral stroke patients. Lower oclusal force in hemi-plegic side with normal denture.
Schimmel, 2010 [3]	Quantitative descriptive study			Masseter muscle	Masseter thickness.	In EG, the masseter muscle in the affected side was thinner than the non-affected side.
Schimmel, 2011a [20]	Non-randomized study			Masticatory efficiency, lip	Two color mixing test. Lip force. OHIP.	Significant difference in chewing efficiency between EG and CG. Maximum lip force was significantly lower in CG. OHRQoL was significantly reduced in stroke patients.
Schimmel, 2011b [22]	Non-randomized study			Teeth, lips	Two color-gum mixing-test. Maximum voluntary bit force. Lip force. Lip seal.	Masticatory efficiency and maximum lip force were significantly reduced in EG. Maximum bite force was not significantly different between both sides and between EG and EC.
Schimmel, 2011c [23]	Non-randomized study			Facial muscles (frontalis, lower facial and orbicularis oris muscles)	Quantitative assessment of facial muscle function. House–Brackmann scale.	Lower facial muscles were more affected than the upper ones, showing muscular weakness in the EG and reduced tonus of the affected orbicularis muscle.
Schimmel, 2013 [24]	Non-randomized study			Teeth, lips, masseter muscle	DMFT. Color-mixing ability test. Maximum restraining lip force. Maximum voluntary bite force. Masseter muscle thickness.	Chewing efficiency: significatly less efficient in stroke patients. Maximum restraining lip force: significantly lower in stroke patients. Maximum voluntary bite force: difference in the cortical control of the jaw closing muscles and those of the upper limb. Masseter muscle thickness: significant difference between contra- and ipsilesional sides, but not between stroke and control groups.
Oh, 2013 [13]	Randomized controlled trial	EG and CG: functional training in their routine rehabilitation EG group: stomatognathic alignment exercise, active ROM exercises for the neck and TMJ	60/3/4	Neck muscles, TMJ	Range of mouth opening. Neck mobility. CMI. MASA.	Significant changes on the opening, CMI, and MASA scores between the EG and CG. Neck mobility: EG showed significant differences between pre- and post-test values in all measures.
Steele, 2016 [25]	A randomized trial	TPSAT: strength targets and accuracy targets TPPT: real saliva swallows	-/2–3/8–12	Tongue	Tongue strength: VFFS.	A significant treatment effect was found in both outcomes. No significant differences between both treatments.
Schimmel, 2017 [26]	Non-randomized study			Lips, tongue, cheeks	Maximum voluntary bite force. Maximum restraining lip force. TDT and 2PD. Color-mixing ability test.	Bite and lip force: no significant difference between both groups. Lower lip force in the stroke group. TDT and 2PD were significantly higher on the affected side in stroke patients. Significantly lower chewing efficiency in stroke patients.
Dursun, 2018 [7]	Non-randomized study			TMJ, masticatory muscles	TMJ ROM. Fonseca questionnaire. Facial asymmetry. PPT of masticatory muscles.	TMJ ROM loss, facial asymmetry, and TMJD were more prevalent in stroke patients. PPT: middle part of the left temporalis muscle was more sensitive to pressure pain in stroke.
Altvater Ramos, 2019 [27]	Quantitative descriptive study			TMJ, temporal, sternocleidomastoid, masseter, and upper trapezius	Mandibular movement. TMJ, facial, and neck muscle PPT ROM cervical region.	81.8% of the patients presented signs and symptoms related to TMD and most of them had a diagnosis of reduced disc displacement. Significant difference in the TMJ and masseter muscle PPT. Cervical ROM: decreased amplitude.
Umay, 2019 [12]	A prospective randomized controlled study	Intermittent galvanic stimulation to masseter muscles and cognitive, sensorimotor and respiratory rehabilitation + Standard dysphagia rehabilitation	60–90/5/4	Tongue, masseter, and orbicularis muscles	Sweep speed and sensitivity of the tongue, masseter, and orbicularis muscles. Electrical activity of submental muscles.	Tongue, masseter, and orbicularis muscles: lower muscle action potential amplitudes. EG presented longer swallowing intervals compared to the healthy control group. Significant improvement in muscle dynamic activity.
Yilzman, 2020 [4]	Quantitative non-randomized study	Active and active assisted ROM exercises for neck and TMJ, chin tuck, breathing and relaxing, and postural exercises + stroke rehabilitation program	5/10/4	TMJ and neck muscles	CMI. Cervical mobility.	All parameters were significantly improved both in 1st and 6th month evaluation.
Choi, 2020 [28]	Open-label, parallel-group, comparative study randomized trial	JOE group: JOE exercises using a resistance bar + traditional dysphagia treatment HLE group: HLE exercises + traditional dysphagia treatment	30/5/6	Digastric and mylohyoid muscles Hyoid bone	Digastric and mylohyoid muscle thickness. Kinematic movement of the hyoid bone.	Both groups showed a statistically significant increase in the thickness of the digastric and mylohyoid muscles and on the anterior and superior movement of the hyoid bone.
Song, 2021 [29]	Non-randomized study			Masseter muscle, masticatory performance	Masseter muscle stiffness and hardness. Masticatory performance.	Masseter muscle hardness and masticatory performance were significantly greater on the unaffected side. A statistically negative moderate correlation between the masseter muscle stiffness of the affected side and the masticatory performance was found.

2PD: two points discrimination threshold; CG: control group; CMI: craniomandibular index; DMFT: decayed, missing, filled teeth; EG: experimental group; HLE: head lift exercises; JOE: jaw opening exercises; MASA: measurements assessment for swallowing ability; OHIP: Oral Health Impact Profile-EDENT; OHRQoL: oral health-related quality of life; PPT: pressure pain threshold; ROM: range of motion; TDT: tactile detection threshold; TMD: temporomandibular disorders; TPPT: tongue-pressure profile training; TPSAT: tongue-pressure strength and accuracy training; VFFS: videofluoroscopy.

**Table 3:** Methodological quality.

Quantitative randomized controlled trials	2.1. Randomization appropriately performed?	2.2. Groups comparable at baseline?	2.3. Complete outcome data?	2.4. Outcome assessors blinded to the intervention provided?	2.5. Participants adhere to the assigned intervention?	% Total
Oh, 2013 [13]	Yes	No	Yes	Yes	No	60%
Steele, 2016 [25]	Yes	Yes	No	Yes	Yes	80%
Umay, 2019 [12]	No	Yes	Yes	Yes	Yes	80%
Choi, 2020 [28]	Yes	Yes	No	No	No	40%
**Quantitative non-randomized**	**3.1. Participants representative of the target population?**	**3.2. Measurements appropriate regarding both the outcome and intervention (or exposure)?**	**3.3. Complete outcome data?**	**3.4. Confounders accounted for in the design and analysis?**	**3.5. Intervention administered (or exposure occurred) as intended?**	**% Total**
kim, 2005 [19]	Yes	Yes	Yes	Yes	Yes	100%
Kawasaka, 2010 [21]	Yes	Yes	Yes	Yes	Yes	100%
Schimmel, 2010 [3]	Yes	Yes	Yes	Yes	Yes	100%
Schimmel, 2011a [20]	Yes	Yes	Yes	Yes	Yes	100%
Schimmel, 2011b [22]	Yes	Yes	No	Yes	Yes	80%
Schimmel, 2011c [23]	Yes	Yes	Yes	Yes	Yes	100%
Schimmel, 2013 [24]	Yes	Yes	No	Yes	Yes	80%
Schimmel, 2017 [26]	Yes	Yes	Yes	Yes	Yes	100%
Dursun, 2018 [7]	Yes	Yes	Yes	Yes	Yes	100%
Yilzman, 2020 [4]	Yes	Yes	Yes	Yes	Yes	100%
Song, 2021 [29]	Yes	Yes	Yes	No	Yes	80%
**Quantitative descriptive**	**4.1. Sampling strategy relevant to address the research question?**	**4.2. Sample representative of the target population?**	**4.3. Measurements appropriate?**	**4.4. Risk of non-response bias low?**	**4.5. Statistical analysis appropriate to answer the research question?**	**% Total**
Altvater Ramos, 2019 [27]	No	No	Yes	Yes	Yes	60%

## Data Availability

Not applicable.
